# Attentional Problems Occur Across Multiple Psychiatric Disorders and Are Not Specific for ADHD

**DOI:** 10.1017/S1092852922000785

**Published:** 2022-05-20

**Authors:** Jon E. Grant, Samuel R. Chamberlain

**Affiliations:** a Department of Psychiatry & Behavioral Neuroscience, University of Chicago; b Department of Psychiatry, Faculty of Medicine, University of Southampton, UK; and Southern Health NHS Foundation Trust, Southampton, UK

**Keywords:** attention, focus, ADHD, impulsivity

## Abstract

**Objective:**

Attentional problems are common and have been associated with multiple psychiatric disorders. This study examined problems of sustained attention across a range of psychiatric disorders using a validated computerized trans-diagnostic attentional paradigm (a Continuous Performance Task). We hypothesized that multiple psychiatric disorders, particularly ADHD, would be associated with pronounced attentional problems in young adults versus controls.

**Methods:**

576 non-treatment seeking participants (aged 18-29 years) were enrolled from general community settings, and provided information regarding demographic variables and underwent clinical assessments to detect a range of mental health disorders. Each participant underwent the Rapid Visual Information Processing (RVP) task, a previously validated computerized test measuring sustained attention. The two measures of sustained attention were the sensitivity index, and target detection (proportion of targets detected). The profile of attentional deficits was examined across different disorders using z-scores relative to controls.

**Results:**

Participants with social phobia, OCD, bulimia nervosa, and intermittent explosive disorder showed the greater impairment in target sensitivity, all with effect sizes of at least 0.8. Target detection was impaired across multiple disorders, with OCD and binge eating disorder exhibiting the most pronounced impairment. PTSD and compulsive sexual behavior were associated with particularly spared performance on both measures.

**Discussion:**

These data indicate that impaired attention is non-specific for ADHD and in fact several other disorders are associated with markedly larger deficits. Instead of clinicians assuming sustained attention problems are due to ADHD, a variety of disorders should be screened for when people report attentional problems. Future work should examine the contribution of comorbidities and psychoactive substances (prescribed or illicit) to the profiles identified.

## Introduction

Attentional problems are common and are associated with multiple psychiatric disorders.^1–6^ Attention deficit hyperactivity disorder (ADHD) may reflect the most extreme version of attentional problems but the complaint of inattention may suggest a disorder other than ADHD. Major depressive disorder, substance use disorders, and a host of anxiety disorders have attentional difficulties as one of their common features.^1–5^ There has been little research, however, regarding the degree to which attention may be problematic across multiple disorders, using objective measures. The issue is important because tests of sustained attention, commonly referred to as Continuous Performance Tasks (CPTs) are seeing increasing use as ‘decision making’ tools in the context of ADHD management.

Although there are a handful of paper-pencil measures by which we can assess attention,^7^ attentional difficulties can also be measured using computer-based instruments such as the Rapid Visual Information Processing (RVP) task, a widely used computerized test of visual sustained attention.^8^ Such tests are not diagnostic of particular disorders. For example, cognitive tests lack sufficient positive predictive power to differentiate ADHD from non-ADHD^9,10^ and are inferior to questionnaires or clinical assessments in this regard^11,12^. Many patients do not have observable deficits on such tasks^11,12^. However, computerized cognitive tasks do have the advantage of being objective, and of being able to quantify distinct cognitive domains likely to be relevant to day-to-day functioning.

Therefore, the aims of this study were to examine sustained attention across a range of psychiatric disorders using this CPT. We hypothesized that multiple psychiatric disorders, particularly ADHD, would be associated with pronounced attentional problems in young adults.

## Methods

### Participants

576 young adult participants (aged 18-29 years) were enrolled from a study in young adults, from general community settings. Study inclusion criteria were: participants had gambled at least five times in the past year (since this was part of a wider longitudinal study that was enriched for gambling); and they were able to be interviewed in person. Exclusion criteria for this study: hearing or vision problems that made performing cognitive tasks difficulty; and an inability to understand and consent to the study. Participants were recruited in the Minneapolis and Chicago metropolitan areas using media advertisements. Each participant received a $50 gift card to an online store as compensation. The Institutional Review Board of the University of Chicago approved the study and the consent statement. The authors assert that all procedures contributing to this work comply with the ethical standards of the relevant national and institutional committees on human experimentation and with the Helsinki Declaration of 1975, as revised in 2008.

### Assessments

Demographic variables, including age, gender, and highest level of education completed, were recorded for all participants. Subjects received a psychiatric evaluation, which included the Mini International Neuropsychiatric Inventory (MINI),^13^ the Minnesota Impulsive Disorders Interview (MIDI) (which screens for compulsive buying, kleptomania, trichotillomania, skin picking disorder, pyromania, intermittent explosive disorder, compulsive sexual behavior, and binge eating disorder),^14–15^ ADHD World Health Organization Screening Tool Part A (ASRS v1.1),^16^ and the Structured Clinical Interview for Gambling Disorder.^17^ For the ADHD definition, in keeping with standard recommendations^16^, endorsement of at least 4 of 6 ADHD symptoms on the ASRS Part A was deemed indicative of this disorder. In the ADHD group, 100% endorsed symptom item 1 to some extent, 98.7% item 2, 98.7% item 3, 96% item 4, 90.7% item 5, and 90.7% item 6. As such, the group can be considered to have the full gamut of ADHD symptom types, as indexed by this tool.

In addition to diagnostic measures, participants underwent computerized testing using the Rapid Visual Information Processing (RVP) task, a test measuring sustained attention that has been widely used in the literature.^8^ RVP is a type of Continuous Performance Test (CPT) paradigm, commonly used in ADHD. For the RVP, participants are requested to detect patterns of number target sequences (e.g., 2-4-6). A white box shown in the center of the screen containing single digits from 2–9 in a random order at a rate of 100 digits per minute. Once the participants see the target sequence they must respond by using the press pad as quickly as possible. Target sequences occurred at the rate of four every 30 s, and the computer calculated both the number of presses to target within a period of 1.5 s after presentation. The sustained attention measures on this task are target sensitivity (a measure of the subject’s ability to distinguish targets and non-targets), and the proportion of targets successfully detected.^18^

### Data Analysis

Only psychiatric disorders endorsed by at least 1% of the participants (i.e. at least 5 people) were included for analysis. The profile of attentional impairment for disorders was quantified by calculating z-scores relative to normative data from those participants in the study who did not have mental disorders (hereafter referred to as controls). Z-scores in this context are equivalent to Cohen’s D and so reflect effect sizes. By convention, 0.3 would be small, 0.5 medium, and 0.8 large effect sizes.

## Results

The sample comprised n=576 participants, mean (standard deviation) age of 22.2 (3.6) years, 377 (65.5%) being female. Of the 576 participants, 424 individuals (73.6%) had completed college education or higher.

**Figure 1:**
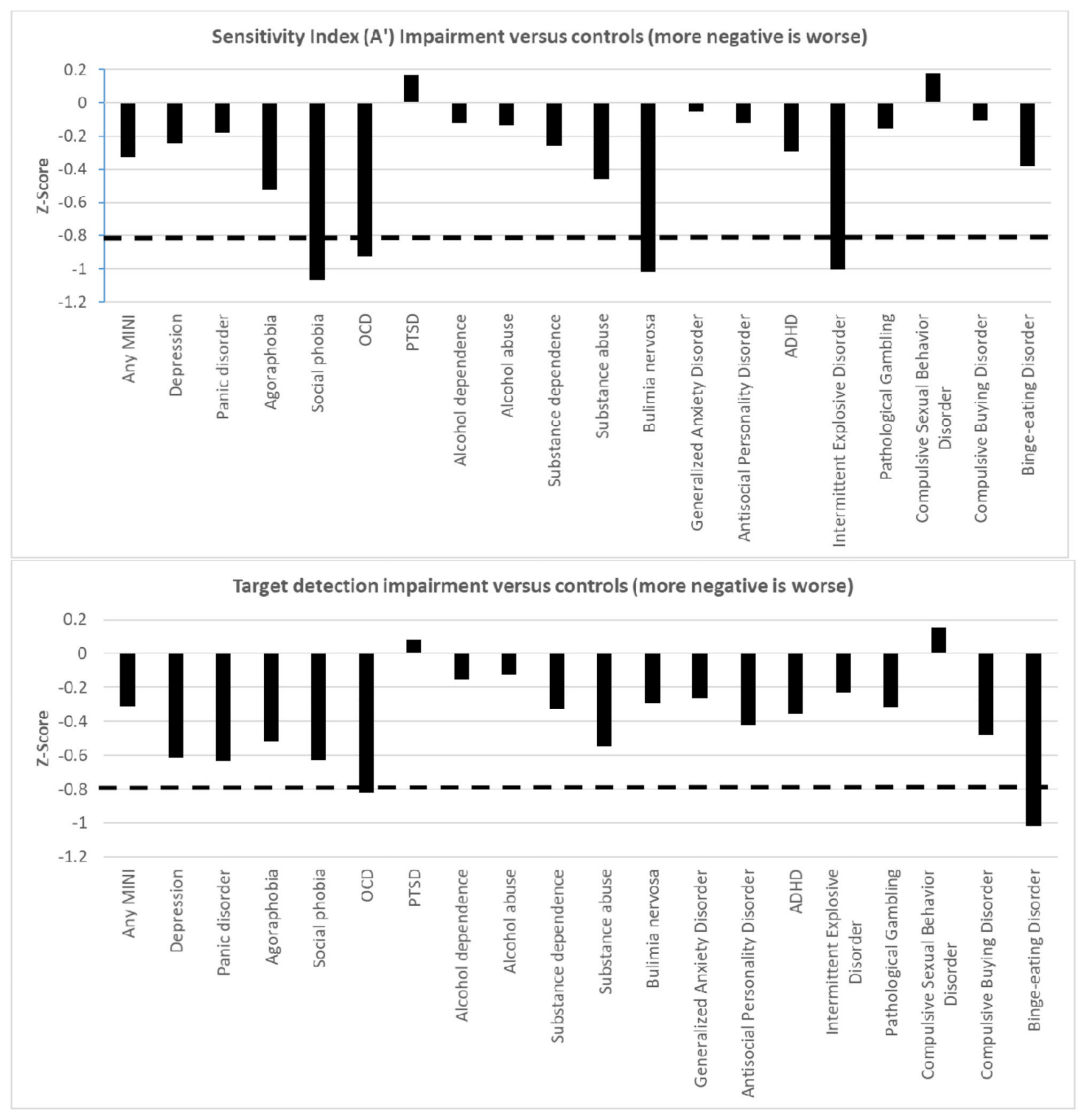
Profile of sustained attention problems across the range of neuropsychiatric disorders. Top panel shows Z-Scores for sensitivity index impairment in patient groups as compared to controls. Lower panel shows Z-Scores for target detection impairment in patient groups as compared to controls. The dotted lines indicate threshold for large effect size deficits (0.8 or higher).

Figure 1 shows the disorders associated with impairment in sensitivity index compared to controls. Those participants with social phobia, obsessive-compulsive disorder (OCD), bulimia nervosa, and intermittent explosive disorder showed the greatest impairment versus controls, all with effect sizes of at least 0.8. Those with probable ADHD exhibited impairment but not to the degree of these other disorders. PTSD and compulsive sexual behavior were both associated with intact (or even superior) sustained attention.

Target detection was impaired across multiple disorders, but OCD and binge eating disorder exhibited the most pronounced impairment (effect sizes of >0.8). PTSD and compulsive sexual behavior were again associated with normal (or even superior) target detection performance (Figure 1).

## Discussion

This is the first study that we are aware of that has used a validated, trans-diagnostic task of sustained attention across multiple disorders in a diverse, non-treatment seeking sample of young adults. This study showed that OCD exhibited the greatest problems of sustained attention, with social phobia and eating disorders (bulimia nervosa and binge eating disorder) exhibiting pronounced difficulties as well. All of these disorders showed greater problems with sustained attention than probable ADHD. These findings may be clinically important as primary doctors and psychiatrics should not assume a diagnosis of ADHD based on a complaint of attentional problems. Based on these data, a chief complaint of attentional problems should elicit a thorough psychiatric examination with a focus on such disorders as OCD, social phobia and eating disorders.

These data may also have clinical including treatment implications. In the case of ADHD, a stimulant is an established first-line pharmacological intervention.^19^ In the case of social phobia or OCD, however, the first-line pharmacological treatment is a selective serotonin reuptake inhibitor (SSRI).^20–21^ Thus, proper diagnosis of the underlying condition giving rise to attention difficulties might result in quite wide ranging differences in treatment. Treatments for most disorders do not focus on sustained attention as a treatment target, notably those associated with the greatest impairment in the current study. Future work should explore the contribution of these deficits to everyday functioning and whether they can be ameliorated in different clinical settings.

Several limitations should be considered. Because this study was conducted in community-recruited participants, the findings may not generalize to clinical populations. For example, clinical populations may perhaps higher levels of attentional problems than observed herein (although large effect size impairments were found in our study for particular disorders, suggesting this may not be a prominent limitation in the current study). Because the study was cross-sectional, direction of effect cannot be shown (i.e. no causal evidence that attentional difficulties lead to these various disorders). Some of the disorders represented have small sample sizes, and so follow-up work in larger samples would be valuable, particularly to more rigorously address links between attention difficulties and the other symptoms defining the various disorders. The cognitive profiles are presented as the patients actually performed on the cognitive task. Co-morbidities are common in all the disorders considered and indeed these disorders often overlap with each other^22^. Psychoactive substance use (illicit or legitimate prescribed medication) use may also be common, though this was a non-treatment seeking sample. The study was neither designed nor powered to address the impact of comorbidities or psychoactive substances on the observed cognitive profiles, and this should be examined in future work, which will require larger sample sizes and different methodological approaches.

## References

[1] Piani MC, Maggioni E, Delvecchio G, Brambilla P (2022). Sustained attention alterations in major depressive disorder: A review of fMRI studies employing Go/No-Go and CPT tasks. J Affect Disord.

[2] Tetik D, Gica S, Bestepe EE, Buyukavsar A, Gulec H (2022). Emotional Information Processing and Assessment of Cognitive Functions in Social Anxiety Disorder: An Event-Related Potential Study. Clin EEG Neurosci.

[3] Kaplan B, Yazici Gulec M, Gica S, Gulec H (2020). The association between neurocognitive functioning and clinical features of borderline personality disorder. Braz J Psychiatry.

[4] Ros-Cucurull E, Palma-Álvarez RF, García-Raboso E, Cardona-Rubira C, Jacas C, Grau-López L, Robles-Martínez M, Daigre C, Ros-Montalbán S, Casas M, Ramos-Quiroga JA (2018). Benzodiazepine Use Disorder and Cognitive Impairment in Older Patients: A Six-Month-Follow-Up Study in an Outpatient Unit in Barcelona. J Stud Alcohol Drugs.

[5] Schachar RJ, Dupuis A, Anagnostou E, Georgiades S, Soreni N, Arnold PD, Burton CL, Crosbie J (2021). Obsessive-compulsive disorder in children and youth: neurocognitive function in clinic and community samples. J Child Psychol Psychiatry.

[6] Pironti VA, Lai MC, Müller U, Dodds CM, Suckling J, Bullmore ET, Sahakian BJ (2014). Neuroanatomical abnormalities and cognitive impairments are shared by adults with attention-deficit/hyperactivity disorder and their unaffected first-degree relatives. Biol Psychiatry.

[7] Treviño M, Zhu X, Lu YY (2021). How do we measure attention? Using factor analysis to establish construct validity of neuropsychological tests. Cogn Research.

[8] Coull JT, Frith CD, Frackowiak RS, Grasby PM (1996). A fronto-parietal network for rapid visual information processing: a PET study of sustained attention and working memory. Neuropsychologia.

[9] Bijlenga D, Ulberstad F, Thorell LB, Christiansen H, Hirsch O, Kooij JJS (2019). Objective assessment of attention-deficit/hyperactivity disorder in older adults compared with controls using the QbTest. Int J Geriatr Psychiatry.

[10] Wasserman T, Wasserman LD (2012). The sensitivity and specificity of neuropsychological tests in the diagnosis of attention deficit hyperactivity disorder. Appl Neuropsychol Child.

[11] Biederman J, Petty CR, Fried R, Black S, Faneuil A, Doyle AE, Seidman LJ, Faraone SV (2008). Discordance between psychometric testing and questionnaire-based definitions of executive function deficits in individuals with ADHD. J Atten Disord.

[12] Lambek R, Tannock R, Dalsgaard S, Trillingsgaard A, Damm D, Thomsen PH (2011). Executive dysfunction in school-age children with ADHD. J Atten Disord.

[13] Sheehan DV, Lecrubier Y, Sheehan KH, Amorim P, Janavs J, Weiller E, Hergueta T, Baker R, Dunbar GC (1998). The Mini-International Neuropsychiatric Interview (M.I.N.I.): the development and validation of a structured diagnostic psychiatric interview for DSM-IV and ICD-10. J Clin Psychiatry.

[14] Grant JE (2008). Impulse control disorders: A Clinician’s guide to understanding and treating behavioral addictions.

[15] Chamberlain SR, Grant JE (2018). Minnesota Impulse Disorders Interview (MIDI): Validation of a structured diagnostic clinical interview for impulse control disorders in an enriched community sample. Psychiatry Res.

[16] Kessler RC, Adler L, Ames M, Demler O, Faraone S, Hiripi E, Howes MJ, Jin R, Secnik K, Spencer T, Ustun TB (2005). The World Health Organization Adult ADHD Self-Report Scale (ASRS): a short screening scale for use in the general population. Psychol Med.

[17] Grant JE, Steinberg MA, Kim SW, Rounsaville BJ, Potenza MN (2004). Preliminary validity and reliability testing of a structured clinical interview for pathological gambling. Psychiatry Res.

[18] Sahakian B, Jones G, Levy R, Gray J, Warburton D (1989). The effects of nicotine on attention, information processing, and short-term memory in patients with dementia of the Alzheimer type. Br J Psychiatry.

[19] Cortese S, Adamo N, Del Giovane C, Mohr-Jensen C, Hayes AJ, Carucci S, Atkinson LZ, Tessari L, Banaschewski T, Coghill D, Hollis C (2018). Comparative efficacy and tolerability of medications for attention-deficit hyperactivity disorder in children, adolescents, and adults: a systematic review and network meta-analysis. Lancet Psychiatry.

[20] Williams T, Hattingh CJ, Kariuki CM, Tromp SA, van Balkom AJ, Ipser JC, Stein DJ (2017). Pharmacotherapy for social anxiety disorder (SAnD). Cochrane Database Syst Rev.

[21] Fineberg NA, Gale TM (2005). Evidence-based pharmacotherapy of obsessive-compulsive disorder. Int J Neuropsychopharmacol.

[22] Geller DA (2006). Obsessive-compulsive and spectrum disorders in children and adolescents. Psychiatr Clin North Am.

